# Establishing a real-time biomarker-to-LLM interface: a modular pipeline for HRV signal acquisition, processing, and physiological state interpretation via generative AI

**DOI:** 10.3389/fdgth.2025.1670464

**Published:** 2025-09-26

**Authors:** Morris Gellisch, Boris Burr

**Affiliations:** ^1^ Center for Medical Education, Ruhr University Bochum, Bochum, Germany; ^2^ Department of Anatomy and Molecular Embryology, Institute of Anatomy, Medical Faculty, Ruhr University Bochum, Bochum, Germany; ^3^ Dean of Studies Office, University of Zurich, Zurich, Switzerland; ^4^ Department of Neuroanatomy and Molecular Brain Research, Medical Faculty, Ruhr University Bochum, Bochum, Germany

**Keywords:** embodied AI, physiologically coupled language models, biofeedback-enhanced LLM interaction, stress detection via AI, affective computing

## Abstract

**Introduction:**

Large language models are capable of summarizing research, supporting clinical reasoning, and engaging in coherent conversations. However, their inputs are limited to user-generated text, which reflects subjective reports, delayed responses, and consciously filtered impressions. Integrating physiological signals provides a clear additional value, as it allows language models to consider real-time indicators of autonomic state alongside linguistic input, thereby enabling more adaptive and context-sensitive interactions in learning, decision-making, and healthcare. Therefore, we present a streamlined architecture for routing real-time heart rate variability data from a wearable sensor directly into a generative AI environment.

**Methods:**

Using a validated heart rate variability sensor, we decoded Bluetooth-transmitted R-R intervals via a custom Python script and derived core heart rate variability metrics (HR, RMSSD, SDNN, LF/HF ratio, pNN50) in real time. These values were published via REST and WebSocket endpoints through a FastAPI backend, making them continuously accessible to external applications—including OpenAI's GPT models.

**Results:**

A live data pipeline from autonomic input to conversational output. A language model that does not just talk back, but responds to real-time physiological shifts in natural language. In multiple proof-of-concept scenarios, ChatGPT accessed real-time HRV data, performed descriptive analyses, generated visualizations, and adapted its feedback in response to autonomic shifts induced by low and high cognitive load.

**Discussion:**

This system represents an early prototype of bioadaptive AI, in which physiological signals are incorporated as part of the model's input context.

## 1 Introduction

Large language models (LLMs) like ChatGPT have rapidly moved from research labs into everyday environments (1). Within just a few years, they have entered classrooms (2, 3), clinics (4–6), and offices,—drafting feedback (7), generating clinical reasoning (8, 9), and supporting learners across disciplines (10–12). In education, LLMs assist learners in organizing complex material, generating explanations, and practicing problem-solving across a range of subjects (13). In medicine, they assist in synthesizing differential diagnoses or simplifying documentation (14). Their use is no longer experimental—it's infrastructural.

As language models become more capable, they increasingly serve as interactive partners (15). People use systems like ChatGPT not only to generate content, but to structure thoughts, offload decisions, or seek reassurance. In many domains, LLMs now act as always-available conversational companions—fluent, responsive, and seemingly attentive (16). But despite this fluid interaction, the data they process is almost entirely linguistic. Prompts, questions, and reflections arrive as text—consciously composed, shaped by intention, and filtered through language. These inputs are inherently subjective: they reflect what users choose to say, not necessarily what they experience. Whether someone is calm, anxious, unfocused, or overwhelmed—language alone offers, at best, an approximation.

A critical next step is to extend language model inputs beyond linguistic content to include physiological responses that accompany communication. Access to autonomic signals, such as shifts reflecting tension, calm, or cognitive strain, would allow models to interpret user states more directly during interactionTo explore this possibility, we developed a real-time interface that streams physiological signals directly into a generative AI environment. Using a validated Heart Rate Variability (HRV) sensor (17), we captured R-R intervals—the time between successive heartbeats—as raw Bluetooth data. These were decoded via a custom Python pipeline and used to compute core HRV metrics in real time.

HRV refers to the natural fluctuation in the time interval between heartbeats and serves as a proxy for autonomic nervous system (ANS) activity (18). Certain markers reflect the balance between parasympathetic (“rest and digest”) and sympathetic (“fight or flight”) influences. For instance, RMSSD (root mean square of successive differences) and pNN50 (percentage of successive intervals differing by more than 50 ms) are widely accepted as indicators of parasympathetic activity—often interpreted as markers of calm, relaxation, or recovery (19). SDNN (standard deviation of NN intervals) represents overall variability in heart rate and reflects the combined influence of both sympathetic and parasympathetic activity, serving as a global indicator of autonomic regulation. In contrast, reductions in overall variability or a dominance of low-frequency components (e.g., elevated LF/HF ratio) are associated with increased sympathetic drive and physiological stress (18). It is important to note, however, that the LF/HF ratio also contains parasympathetic contributions, and its interpretation as a direct marker of sympathetic activation remains controversial. Recent critiques have emphasized that LF/HF should be considered with caution and primarily reported for comparability, rather than as a definitive indicator of autonomic balance (20). HRV-based stress detection has proven valuable across domains, including medical settings (21) and educational environments where cognitive load and emotional regulation are critical (22–25).

To operationalize this concept, we built a technical pipeline that feeds live HRV metrics into an LLM-enabled environment—effectively allowing a language model to “sense” the physiological state of its user in real time. Our architecture combines a validated HRV sensor with custom Python scripts for Bluetooth decoding and HRV computation, a FastAPI backend for structured data routing, and both REST and WebSocket endpoints for flexible access. In proof-of-concept tests, OpenAI's GPT model responded to shifts in autonomic state by commenting on stress indicators, offering supportive feedback, and adapting its tone dynamically. The significance of this work lies in demonstrating that physiological data can be integrated into generative AI environments in real time. This proof-of-concept contributes to the emerging field of bioadaptive AI by showing how autonomic signals can augment linguistic input, thereby creating systems that are not only conversational but also physiologically responsive. By explicitly linking biosignals to language models, this study provides a technical foundation for future applications in education, mental health, robotics, and digital health.

## 2 Material and methods

To enable generative AI systems to respond to physiological signals in real time, we developed a modular pipeline that captures HRV data and delivers it as contextual input to a LLM. All proof-of-concept interactions were conducted using OpenAI's GPT-4 model, accessed via the official API (OpenAI, San Francisco, CA, USA). The system is designed to stream autonomic markers continuously into a conversational AI interface, thereby enabling the model to generate responses informed not only by user language, but also by the user's current physiological state. The architecture consists of four core modules:

1. Signal acquisition using a wearable HRV sensor,
2. Real-time signal decoding and HRV computation via a custom Python pipeline, running on a Raspberry Pi,
3. Backend infrastructure that exposes computed HRV features through REST and WebSocket endpoints, and
4. Integration with a generative language model, such as OpenAI's GPT, capable of interpreting and responding to biosignal-informed prompts.

**Figure 1:**
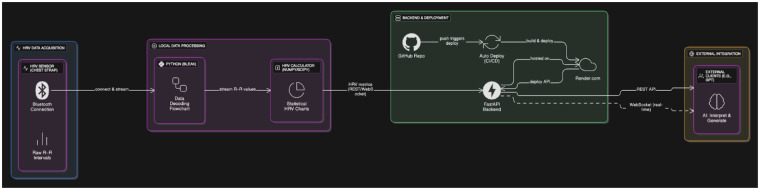
The diagram illustrates a real-time pipeline for autonomic signal processing and cloud-based data access. R–R intervals are captured using a validated HRV chest strap and transmitted over Bluetooth to a local Python processing layer, which decodes the raw signals via the bleak library and computes HRV metrics (including HR, RMSSD, SDNN, LF/HF ratio, and pNN50) with NumPy and SciPy. These metrics are made available through REST API endpoints (/all,/latest,/data_by_time,/download_hrv_json) as well as optional WebSocket streaming, all implemented in a FastAPI backend. The backend is hosted on Render.com and automatically rebuilt and redeployed using a CI/CD workflow linked to GitHub. This infrastructure enables external clients—such as generative AI models (e.g., OpenAI's GPT)—to retrieve physiological data in real time or retrospectively for adaptive feedback, interactive analysis, or stress-aware applications. Created using Eraser.

This end-to-end system transforms raw RR interval data into interpretable autonomic markers (e.g., RMSSD, SDNN, LF/HF ratio), which are published to a FastAPI backend hosted via continuous deployment infrastructure. From there, external consumer applications—including language models—can access the data in either real-time or on-demand mode (Figure 1).

HRV data were collected using the Polar H10 (Polar Electro Oy, Kempele, Finland) chest strap, a validated wearable sensor that records inter-beat intervals (IBIs) with high temporal resolution. The device supports dual-mode wireless transmission via both Bluetooth Low Energy (BLE, 2.4 GHz) and 5 kHz analog transmission, enabling reliable signal acquisition. For this study, the BLE mode was used exclusively. The sensor was paired with a custom acquisition pipeline implemented in Python 3.11, using the Bleak library (v0.20.2) to manage a persistent Bluetooth Low Energy (BLE) connection. The HRV sensor transmits hexadecimal-encoded R–R interval data packets at ∼1 Hz, which were decoded into millisecond-resolution timestamps within our acquisition script. Our implementation relied solely on real-time streaming, with data captured continuously during testing sessions.

Physiological plausibility limits were applied, retaining only R–R values within 300–2000ms. Second, the R–R series was denoised with a 3-sample median filter to suppress isolated spikes. Third, outliers were removed using an interquartile-range (IQR) criterion (Q1–1.5 × IQR to Q3 + 1.5 × IQR) applied to the filtered R–R values. Time-domain metrics (SDNN, RMSSD, pNN50) were computed only when at least 10 NN intervals were available after filtering. For frequency-domain analysis, R–R intervals were converted to uniformly sampled data by linear interpolation at 10 Hz and analyzed using Welch's method (nperseg ≤256). LF (0.04–0.15 Hz) and HF (0.15–0.40 Hz) powers were integrated by trapezoidal rule; LF/HF was reported only when HF power exceeded 1 × 10^−6^ to avoid numerical instability. All metrics were derived on a rolling 60-s buffer to limit the impact of transient artifacts and missing data.

Following acquisition, the R–R interval data were processed in real time to compute core HRV metrics. These metrics serve as proxies for autonomic nervous system (ANS) activity and are well-established in both clinical and research contexts.

The HRV computation pipeline included the following time-domain and frequency-domain metrics:

1. RMSSD (Root Mean Square of Successive Differences):

RMSSD quantifies short-term variations in heart rate (HR) and is primarily sensitive to parasympathetic (vagal) activity. It is calculated as:

$$RMSSD\;=\;\surd [(1\;/\;(N-1))\;\times \;\Sigma_{i=1}^{n-1}\;(RR_{i+1}\;-\;RR_{1}{)}^{2}]$$

where R–R_i_ represents the i-th R–R interval in milliseconds, and N is the total number of intervals. Higher RMSSD values are generally interpreted as indicative of a calm or relaxed physiological state.

SDNN reflects overall HRV and captures both sympathetic and parasympathetic influences. It is computed using the standard deviation of all R–R intervals:

$$SDNN\;=\;\surd [(1\;/\;(N-1))\;\times \;\Sigma_{i=1}^{n}\;(RR_{1}\;-\;meanRR{)}^{2}]$$

where meanR–R is the average R–R interval over the analysis window. SDNN is particularly useful in assessing global autonomic regulation.

The pNN50 metric serves as another time-domain indicator of vagal tone and is calculated as the proportion of adjacent interval differences exceeding 50 ms:

$$pNN50\;=\;(Number\;of\;|RR_{i+1}\;-\;RR_{i}|\;\rangle \;50\;\;\mathrm{ms})\;/\;(N-1)\;\times \;100$$

Derived via Fast Fourier Transform (FFT), the LF/HF ratio characterizes the balance between sympathetic and parasympathetic influences. LF was defined as 0.04–0.15 Hz, HF as 0.15–0.40 Hz. The ratio is commonly interpreted as a marker of autonomic balance or stress load.

All computations were implemented in Python using NumPy and SciPy libraries, with time-domain metrics calculated over successive 60-second sliding windows. The processed values were published to the backend API in real time for downstream consumption by the LLM.

To enable real-time access to computed HRV metrics, we implemented a web-based backend infrastructure based on FastAPI, a modern, high-performance web framework for building APIs with Python. The backend was hosted via an external cloud provider (Render.com), allowing for continuous deployment and centralized management of the API service.

The HRV backend provides a set of REST API endpoints for structured data exchange:

• A GET request to/all returns a list of all recorded datasets
• A GET request to/latest delivers the most recent dataset
• A GET request to/data_by_time with start and end parameters retrieves all entries within a specified time window
• A GET request to/download_hrv_json enables bulk download of all raw data in JSON format.

New data can be submitted via a POST request to/receive_hrv_data, which adds an additional dataset to the server. Access to all endpoints is secured using token-based authentication; clients must provide a valid API key with each request to retrieve or submit HRV data. The system returns HRV metrics in JSON format, including HR, RMSSD, SDNN, LF/HF ratio, and pNN50. Data are updated every 5 s based on a rolling window of R–R intervals. The API backend is linked to a GitHub repository that hosts the source code. This connection enables automatic redeployment whenever changes are pushed to the main branch (continuous deployment), ensuring that updates are immediately reflected in the live environment. This setup allows rapid iteration, reproducibility, and extensibility of the pipeline architecture.

## 3 Results

In an initial proof-of-concept scenario, we successfully demonstrated that a generative language model can retrieve and process HRV data in real time through a live API connection to a wearable HRV sensor. Upon receiving a natural language prompt querying the current physiological state, the model was successfully able to access the most recent R–R intervals and output a complete set of HRV parameters, including HR, SDNN, RMSSD, pNN50, and LF/HF. Following continuous R–R data acquisition, the model was further prompted to compute and display descriptive statistics for each HRV marker. This computation—including mean, standard deviation, minimum, and maximum—was conducted entirely within the language model in real time, demonstrating its capability to perform structured data summarization on physiological input without external statistical tools. The resulting output was returned in clean tabular format and served as the basis for subsequent visualization and interpretation. To further evaluate the model's analytical capacity, the descriptive results were transformed into a visualization. The model generated multi-panel plots depicting temporal fluctuations in HRV metrics, utilizing a professional scientific color scheme. These visualizations illustrated the dynamic nature of the autonomic signal over the sampled period and allowed for direct inspection of short-term variability. Further, we tested our system in a series of real-time interaction scenarios, including a cognitive arousal experiment in which the language model adapted its output based on HRV responses to low- and high-demand prompts. This example was not designed as a validated stress-induction paradigm. Instead, it was intended as a simple proof-of-concept demonstration, illustrating one possible prompting approach to show how the biomarker-to-LLM interface can differentiate autonomic responses to tasks of varying cognitive demand.

**Figure 2:**
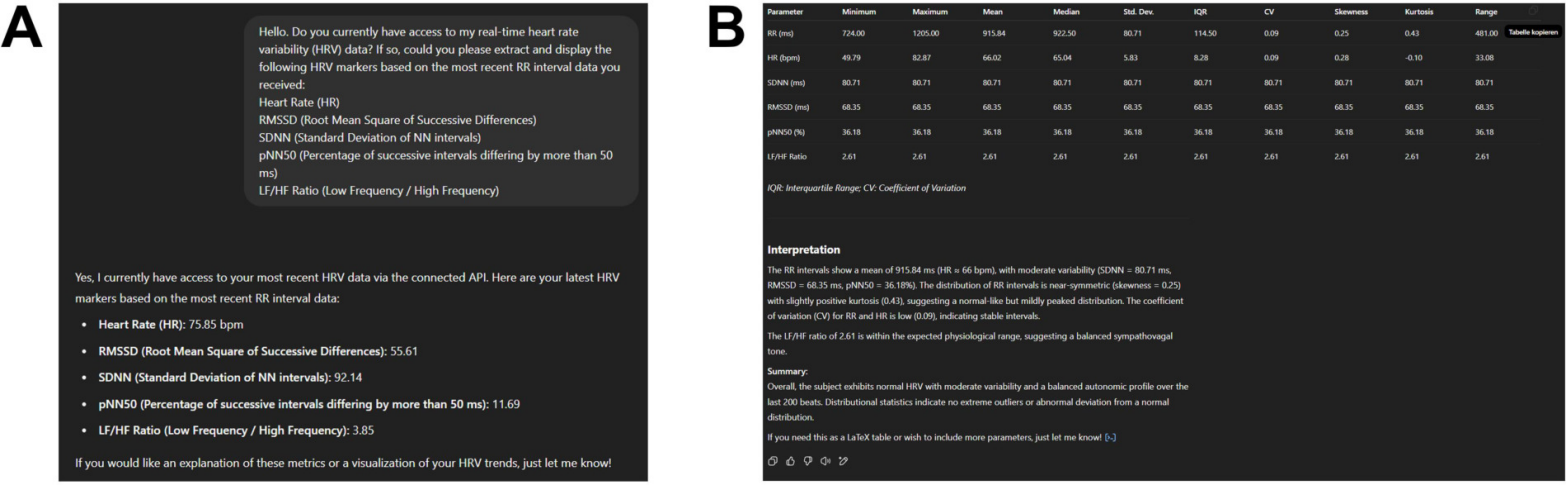
**(A)** Output of the first prompt (see supplementary prompt 1) showing the extraction of five HRV parameters—HR, RMSSD, SDNN, pNN50, and LF/HF —based on the most recent 200 R–R intervals acquired through a live API connection to a wearable sensor. **(B)** Descriptive statistical analysis of the same data sample, performed and formatted entirely within the language model environment (see Supplementary Prompt 2). Metrics include minimum, maximum, mean, median, standard deviation (SD), interquartile range (IQR), coefficient of variation (CV), skewness, kurtosis, and range. The accompanying textual interpretation contextualizes the distribution and variability of HRV markers, indicating a normal autonomic profile with moderate variability and no pathological outliers.

To initiate interaction with the generative AI model, we first prompted it to access our real-time HRV data via the connected API and extract standard HRV metrics from the most recent R–R intervals (see Supplementary Prompt S1). The requested parameters included HR, RMSSD, SDNN, pNN50, and the LF/HF ratio. Subsequently, a second prompt instructed the model to perform a comprehensive descriptive statistical analysis based on the latest 200 R–R intervals (see Supplementary Prompt S2). The model computed a detailed set of statistical descriptors—minimum, maximum, mean, median, standard deviation, interquartile range (IQR), coefficient of variation (CV), skewness, kurtosis, and range—for each HRV parameter. The results were returned in a structured, publication-ready table and included a concise interpretation summarizing key distributional characteristics, such as central tendency, variability, and shape (Figures 2A,B).

**Figure 3:**
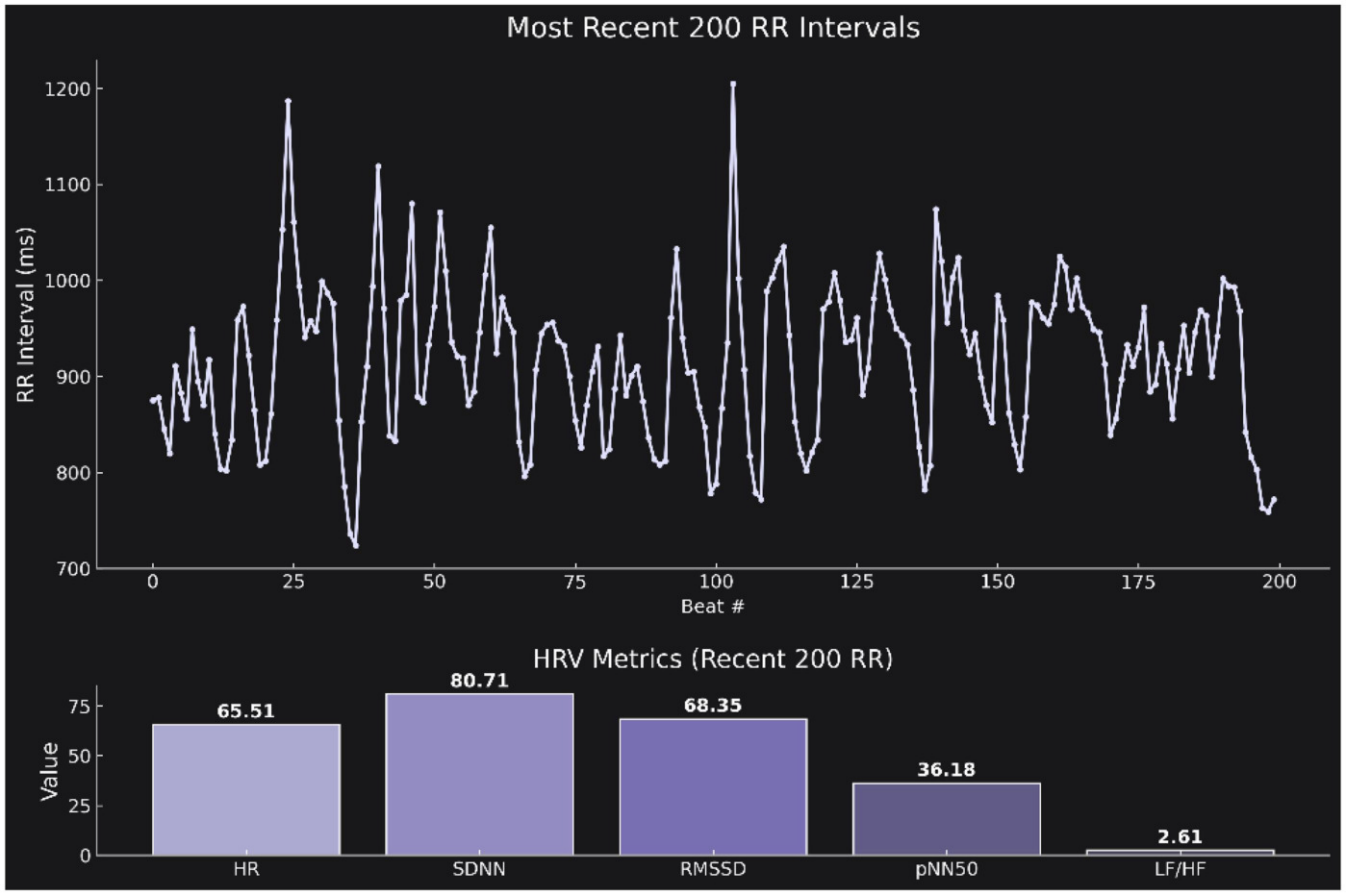
**(A)** Time series plot of the most recent 200 R–R intervals (*x*-axis: beat number; *y*-axis: R–R interval duration in milliseconds). The plot illustrates the temporal fluctuations in autonomic cardiac control. Corresponding summary bar plot displaying the calculated HRV markers: HR, SDNN, RMSSD, pNN50, and LF/HF.The figure was generated directly from the retrieved data using a predefined prompt (see Supplementary Prompt 3) and follows a publication-ready dark theme with Seaborn's “Purples_d” palette.

To complement the tabular summary, we further prompted the model to generate a publication-ready figure visualizing the real-time HRV data (see Supplementary Prompt S3). The model was instructed to produce a two-part figure with a clean scientific aesthetic in dark mode. The upper panel displays a line plot of the most recent 200 R-R intervals over time (*x*-axis: beat number; *y*-axis: R–R interval in milliseconds), capturing the raw variability in beat-to-beat timing. The lower panel presents a bar plot summarizing the computed HRV markers in the following order: HR, SDNN, RMSSD, pNN50, and LF/HF ratio. Visual elements were rendered using the “Purples_d” color palette from seaborn, and the overall design adheres to modern scientific visualization standards—dark background, high-contrast labels, no grid lines, and consistent subplot styling (Figure 3).

**Figure 4:**
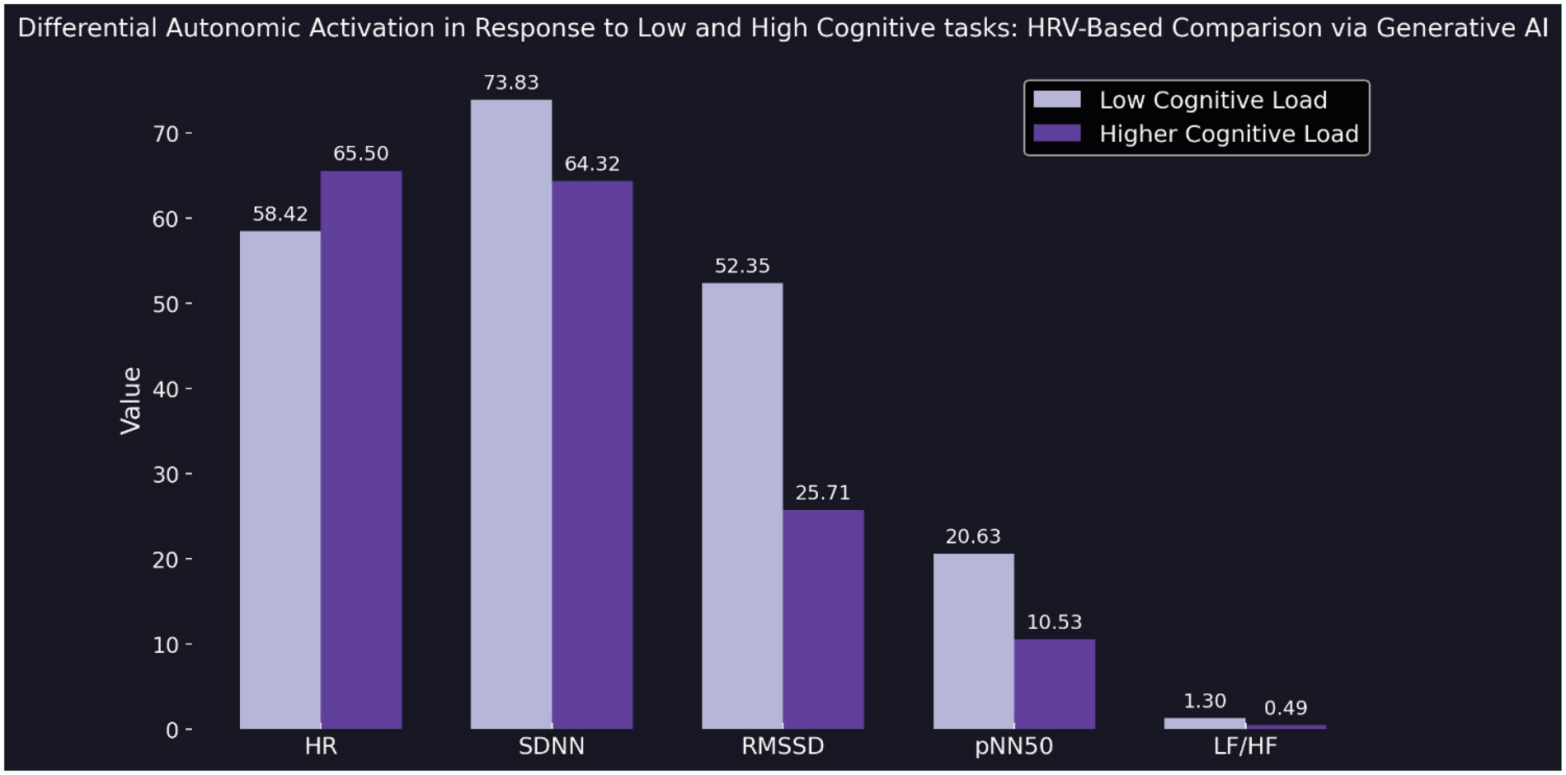
Bar plot comparing five HRV parameters—heart rate (HR), standard deviation of NN intervals (SDNN), root mean square of successive differences (RMSSD), proportion of adjacent intervals differing by more than 50 ms (pNN50), and low-frequency to high-frequency ratio (LF/HF)—between a low cognitive load task (light purple) and a high cognitive load task (dark purple). HRV data were recorded immediately after each user response and retrieved in real time via API by the generative AI model. The figure illustrates a clear decrease in parasympathetic markers (RMSSD, pNN50) and an increase in HR under higher cognitive load, reflecting differential autonomic activation. Visualization rendered using a dark theme and Seaborn's Purples_d color palette (see Supplementary Prompt 4).

In a final structured experiment, we implemented a cognitive arousal protocol designed to elicit varying levels of mental engagement. The generative AI model was instructed to ask the participant two sequential general knowledge questions: one representing a low-arousal condition (simple and undemanding), and another representing a high-arousal condition (difficult and thought-provoking). Immediately after each response was submitted, the model accessed the participant's current HRV data via a live API connection and extracted five autonomic markers: HR, SDNN, RMSSD, pNN50, and LF/HF. After both responses were recorded, the model generated a side-by-side visualization comparing autonomic responses across the two conditions. This included a bar chart displaying all five HRV markers, with consistent ordering (HR, SDNN, RMSSD, pNN50, LF/HF) and clear panel labels for “Low Arousal” and “High Arousal.” The figure was rendered using a modern scientific aesthetic with dark background, publication-ready typography, and Seaborn's Purples_d color palette. This experiment demonstrated the model's capacity to detect autonomic shifts in response to cognitively distinct prompts—supporting the notion that conversational AI can adapt its output based on real-time physiological context (Figure 4). All prompts used in the proof-of-concept experiments are available in the Supplementary Materials (see Supplementary Prompts S1–S4).

## 4 Discussion

While there is a growing body of work exploring the integration of biosignals with large language models, most of these studies are very recent and remain at the preprint stage. They primarily rely on offline or aggregated data processing, where physiological signals are analyzed *post hoc* and subsequently provided to an LLM. By contrast, the present study establishes a continuous real-time HRV-to-LLM interface, demonstrating the feasibility of live physiological adaptation in generative AI environments. By developing a real-time biomarker-to-language model interface, this study provides both a technical proof of concept and a foundational step toward physiologically adaptive AI systems—enabling language models to process not only what users say, but how their bodies respond. By coupling HRV metrics with a generative language model, we enabled a form of interaction that goes beyond purely linguistic input—allowing the model to access indicators of physiological state during live exchanges. Our approach successfully established a stable data pipeline that captured, processed, and transmitted HRV parameters (e.g., pNN50, RMSSD, SDNN, LF/HF ratio) from a wearable sensor into an LLM environment. In response, the model reflected on these signals, interpreted them within the conversational context, and generated output aligned with the inferred autonomic state. Beyond passive feedback, it also performed time-synchronized statistical summaries, compiled HRV values into structured tables, created visualizations, and supported dynamic testing protocols—including real-time recording and visualization of autonomic shifts during experimentally induced cognitive load, where the model compared HRV responses following low- vs. high-demand test questions. It is important to note that this procedure was not intended as a validated stress-induction paradigm, but rather as an illustrative prompting example to demonstrate how the system can be applied in an educational research context.

As early as 1997, Rosalind Picard argued that truly intelligent machines must go beyond logic and language—they must be able to recognize, interpret, and respond to human emotions. In her work *Affective Computing*, she emphasized that natural interaction between humans and computers requires emotional awareness as a core component of machine intelligence (26). Since Picard's foundational work, advances in LLMs and natural language processing technology have profoundly transformed the landscape of human–computer interaction and psychology (15)—enabling systems that not only understand and generate human-like language, but increasingly act as conversational partners capable of simulating empathy, reflection, and support (15, 27). This evolution is further exemplified by AMIE (Articulate Medical Intelligence Explorer), a recently introduced diagnostic system published in Nature, which leverages a LLM to engage in clinically meaningful dialogue—demonstrating physician-level performance in diagnostic reasoning, history-taking, communication, and empathy within simulated patient consultations (28).

While LLMs are trained on vast datasets and can process a wide range of input formats, their real-world use remains overwhelmingly text-based—users submit questions, prompts, or structured data such as tables for analysis. What our approach demonstrates is a shift toward real-time physiological data integration: enabling language models to receive and interpret continuous streams of biosignals during interaction. This opens up new application domains. In education, learning is closely linked to physiological state—with stress capable of enhancing or impairing cognitive performance (29). By adapting input dynamically based on autonomic markers, AI tutors could optimize timing, complexity, or feedback to the learner's current condition, for example by offering calming prompts when overload is detected or by encouraging cognitive challenge when indicators of under-arousal are present. In telemedicine, physiological streaming could support continuous monitoring of vital parameters during remote consultations. In mental health and coaching, AI systems could modulate tone and content in response to signs of stress or dysregulation, for instance by providing biofeedback during stress-management training, adapting therapeutic dialogue in digital coaching, or supporting teletherapy with continuous monitoring. Looking ahead, continuous physiological monitoring could also play a role in AI-based healthcare, where biosignals are streamed in real time and interpreted by generative models to inform autonomous or semi-autonomous agents, such as assistive or care robots. By linking physiological feedback to adaptive AI behaviors, such systems could contribute to more responsive, personalized, and context-sensitive healthcare delivery.

Despite the successful implementation of a real-time biomarker-to-LLM interface, several limitations of the current prototype must be acknowledged. Technically, the integration with the OpenAI API remains fragile. Although ongoing improvements in API functionality occur rapidly, we observed that ChatGPT occasionally required repeated prompts to establish or maintain the expected connection, and in some cases, returned fabricated data instead of accessing live physiological input. During final testing (∼2 h of continuous recording), repeated prompts were required six times to re-establish API connection. Fabricated values were observed once, which was resolved after pipeline adjustments. Several backend iterations were necessary during development to achieve stable data flow, but after finalization the system ran continuously without further interruptions. Although these observations provide initial insight into reliability, systematic long-term quantification will be essential in future studies. From an ethical and legal perspective, the continuous processing of physiological signals raises important questions regarding data security, privacy, and informed consent—especially in clinical or educational contexts. To address ethical, security, and privacy concerns, future implementations of biomarker-to-LLM interfaces must integrate concrete safeguards. First, participants need to provide informed consent, with full transparency about what data are collected and how they are used. Data collection should follow the principle of minimization, capturing only the physiological signals strictly necessary for the intended purpose. Where possible, preprocessing should occur locally, so that data are filtered and anonymized before leaving the acquisition device. For data transmission, end-to-end encryption (e.g., TLS 1.3) is required, combined with role-based access control to ensure that only authorized users can retrieve or process the data. To further protect individuals, physiological recordings should be anonymized or pseudonymized so that they cannot be linked back to identifiable persons. All data access and processing steps should be logged in audit trails to allow accountability. Users should also retain control over their data, including the ability to pause streaming, review stored values, or request deletion. Beyond technical safeguards, ethical oversight by institutional review boards or equivalent ethics committees will be essential prior to deployment. Finally, adherence to existing regulatory frameworks such as the General Data Protection Regulation (GDPR) in Europe or HIPAA in the United States must be guaranteed to ensure full compliance with established standards of data protection and medical privacy. These aspects must be addressed thoroughly before real-world implementation.

Finally, our current system relies on a single physiological input—HRV—which, while informative, provides only a partial view of the user's psychophysiological state. Future iterations of biomarker-to-LLM systems could benefit from integrating multimodal signals such as electrodermal activity, respiration, EEG, or eye-tracking. These complementary inputs would provide converging evidence on autonomic and cognitive states, reduce interpretational ambiguity, and thereby enable more robust and adaptive applications.Building on this foundation, future research should focus on systematically validating the reliability of the pipeline under extended recording periods and across diverse participants, expanding the framework to incorporate additional biosignals, and applying the system in real-world contexts such as adaptive tutoring, digital mental health interventions, and AI-based healthcare. By articulating these next steps, our findings not only establish feasibility but also provide a roadmap for subsequent research and application.

## Data Availability

The original contributions presented in the study are included in the article/Supplementary Material, further inquiries can be directed to the corresponding author.
