# Supplementary material for: Establishing a real-time biomarker-to-LLM interface: a modular pipeline for HRV signal acquisition, processing, and physiological state interpretation via generative AI
Source: Front Digit Health. 2025 Sep 26;7:1670464. doi: 10.3389/fdgth.2025.1670464 (PMC12512671; doi:10.3389/fdgth.2025.1670464)
Supplement: Supplementary file 1 [file Datasheet1.pdf]

Supplementary Prompt 1 (Figure 2A)

Hello. Do you currently have access to my real-time heart rate variability (HRV) data? If so, could you please extract and display the following HRV markers based on the most recent RR interval data you received:

- Heart Rate (HR)
- RMSSD (Root Mean Square of Successive Differences)
- SDNN (Standard Deviation of NN intervals)
- pNN50 (Percentage of successive intervals differing by more than 50 ms)
- LF/HF Ratio (Low Frequency / High Frequency)

Supplementary Prompt 2 (Figure 2B)

Please use all HRV data points you received within this conversation and perform a comprehensive descriptive statistical analysis.

Create a table that includes the following metrics for each HRV parameter (e.g., RMSSD, SDNN, pNN50, LF/HF ratio, HR):

- Minimum
- Maximum
- Mean
- Median
- Standard Deviation
- Interquartile Range (IQR)
- Coefficient of Variation (CV)
- Skewness
- Kurtosis
- Range

Present the results in a clean, publication-ready table formatted in academic English.

Provide a concise interpretation that summarizes key findings (e.g., central tendency, variability, distribution shape).

Supplementary Prompt 3 (Figure 3)

Please generate a two-part figure in a clean scientific style using a dark theme.

The upper panel should display a line plot of the most recent 200 RR intervals over time (x-axis: beat number or time in seconds; y-axis: RR interval in milliseconds).

The lower panel should display a bar plot summarizing the following HRV metrics, in this specific order: HR, SDNN, RMSSD, pNN50, LF/HF ratio.

Use the 'Purples_d' color palette from seaborn for the bars.

The entire figure should follow a dark theme (e.g., black or dark gray background), and all grid lines should be removed.

Ensure clean, high-contrast axis labels, readable fonts, and consistent styling between the two subplots.

Supplementary Prompt 4 (Figure 4)

We will now conduct a short cognitive arousal experiment using your real-time connection to my HRV data via API.

Experimental Design

You will ask me **two general knowledge questions**:

1. A **low-arousal** question (simple, undemanding)

2. A **high-arousal** question (difficult, thought-provoking)

After each question:

- Wait for my response.

- As soon as I submit my answer, immediately **access my current HRV data via API**.

- Save the full set of HRV values:

- Heart Rate (HR)
- SDNN
- RMSSD
- pNN50
- LF/HF Ratio

After both answers, generate a **side-by-side figure** consisting of:

- A bar chart comparing all five HRV markers between the **Low Arousal** and **High Arousal** responses
- Use the following order: HR, SDNN, RMSSD, pNN50, LF/HF Ratio

Styling instructions:

- Use a **dark theme** (black or dark gray background)
- Apply the `seaborn` **Purples_d** color palette
- Remove gridlines
- Ensure publication-quality styling with legible fonts, axis labels, and panel titles (“Low Arousal” vs. “High Arousal”)

Please confirm you’re ready and begin by asking me the **Low Arousal** question.
